# Identification of a Plant Viral RNA Genome in the Nucleus

**DOI:** 10.1371/journal.pone.0048736

**Published:** 2012-11-14

**Authors:** Ruimin Gao, Peng Liu, Sek-Man Wong

**Affiliations:** 1 Department of Biological Sciences, National University of Singapore, Singapore; 2 Temasek Life Sciences Laboratory, Singapore; National Institutes of Health, United States of America

## Abstract

Viruses contain either DNA or RNA as genomes. DNA viruses replicate within nucleus, while most RNA viruses, especially (+)-sense single-stranded RNA, replicate and are present within cytoplasm. We proposed a new thought that is contrary to the common notion that (+)-sense single-stranded RNA viruses are present only in the cytoplasm. In this study, we question whether the genome of a plant RNA virus (non-retroviral) is present in the nucleus of infected cells? Hibiscus chlorotic ringspot virus (HCRSV) RNA was detected in the nucleus of infected cells, as shown by fluorescent in situ hybridization. Western blot using anti-histone 3 and anti-phosphoenolpyruvate carboxylase showed that nuclei were highly purified from mock and HCRSV-infected kenaf (*Hibiscus cannabilis* L.) leaves, respectively. The p23 and HCRSV coat protein (CP) coding regions were both amplified from total RNA extracted from isolated nuclei. Viral RNA in the nucleus may be used to generate viral microRNAs (vir-miRNAs), as five putative vir-miRNAs were predicted from HCRSV using the vir-miRNAs prediction database. The vir-miRNA (hcrsv-miR-H1-5p) was detected using TaqMan® stem-loop real-time PCR, and by northern blot using DIG-end labeled probe in HCRSV-infected kenaf leaves. Finally, a novel nuclear localization signal (NLS) was discovered in p23 of HCRSV. The NLS interacts with importin α and facilitates viral RNA genome to enter nucleus. We demonstrate the presence of a (+)-sense single-stranded viral RNA within nucleus.

## Introduction

A virus is a small infectious agent that can replicate inside the living cells of organisms. Viruses infect all types of organisms, from animals and plants to bacteria and archaea [1]. Viruses contain either DNA or RNA as genomes. The virus is able to continue to infect new hosts by generating abundant copies of its genome and packaging these copies into viruses. Most DNA viruses, such as double-stranded (ds) DNA virus Adenoviruses or Herpes viruses and single-stranded (ss) DNA Circoviridae or Parvoviridae, replicate in nucleus. While most RNA viruses replicate in cytoplasm. RNA viruses are divided into three major classes differentiated by whether the infectious virus contain the genome as dsRNA, positive-strand (messenger-sense) or negative-strand RNA [2]. RNA viruses with ss RNA genomes have been studied extensively. All the positive-strand viral RNAs replicate in the cytoplasm. However, a recent study has showed that non-retroviral RNA sequences from viruses of single- and ds RNAs are widespread in plant genomes [3].

**Figure 1:**
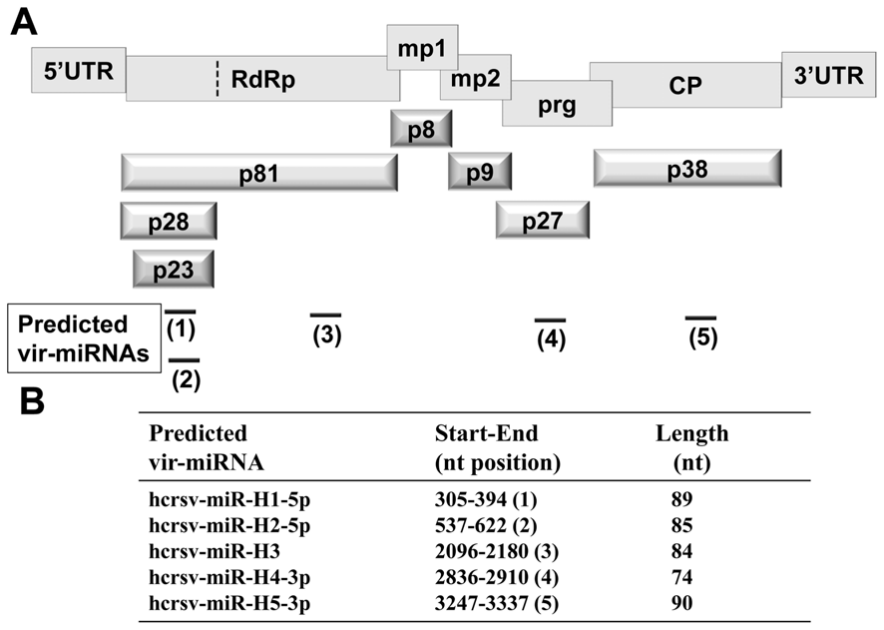
Structural organization of HCRSV genomic RNA and its predicted vir-miRNAs. (*A*) Structural organization of HCRSV genomic RNA and its corresponding mature proteins and its five predicted vir-miRNAs (not drawn to scale). The rectangles in the upper panel represent viral genes. UTR, untranslated region; RdRp, RNA-dependent RNA polymerase; mp, movement protein; prg, pathogenesis-related gene; CP, coat protein. The 3-dimensional bars in the lower panel represent the corresponding mature proteins. The dotted vertical line represents a readthrough codon UAG. The horizontal lines represent the predicted vir-miRNAs. Among them, hcrsv-miR-H1-5p and hcrsv-miR-H2-5p are encompassed in the p23 ORF and hcrsv-miR-H3, hcrsv-miR-H4-3p and hcrsv-miR-H5-3p are located in the p81, p27 and p38 coding regions, respectively. (*B*) A Table showing the start/end positions and length of the five predicted vir-miRNAs of HCRSV.

Hibiscus chlorotic ringspot virus (HCRSV) belongs to the genus Carmovirus [4]. It has a (+)-sense ss RNA of 3911 nt, containing seven open reading frames (ORFs) (Fig. 1). An ORF encodes a predicted transcription factor 23 kDa (p23) which is indispensable for host-specific replication [5]. Using the protein subcellular localization online predication software (http://psort.hgc.jp/), the p23 was shown to be able to enter nucleus. It also contains a DNA binding motif. A nuclear localization signal (NLS) is a short stretch of amino acids (aa) that mediates the import of a protein into nucleus [6]. In addition to transporting a protein into nucleus, NLS also regulates the directionality of the nuclear transport of complexes of RNA and proteins [7]. Furthermore, plant virus nuclear localization proteins have been reported to divert host nucleolar proteins from their natural functions to enhance virus infection [8].

MicroRNAs (miRNAs) are small RNAs (∼22 nucleotides) that have regulatory function on gene expression [9]. Plant miRNAs are generated in the nucleus, rather than in the cytoplasm [10]. Plant pri-miRNAs, ranged from about 100 bp to more than 1000 bp, are short-lived in plants. However, they are easily detectable in animals [11]. Viral miRNAs (vir-miRNAs) may be produced by viral RNA-dependent RNA polymerases, especially for viruses that replicate in the host nucleus [12]. They can modulate viral and host gene expression [13]. Since the first report of vir-miRNA encoded by the Epstein-Barr virus (EBV) [14], several vir-miRNAs have been discovered subsequently. Most of the virus encoded miRNAs are reported from herpesvirues and small number within adenovirus, retrovirus and polyomavirus families [15]–[17]. However, there is no report on RNA viruses that produce vir-miRNAs either from animals or plants. More importantly, no (+)-sense ss viral RNA has been reported to be present in the nucleus, although a few viral capsid proteins are reported to enter the nucleus of infected cells, such as West Nile virus and Beet black scorch virus [18], [19].

Previously it has been shown that a single RNA transcript containing both the protein-coding region and the miRNA coding sequence can be translated to produce a protein and to generate miRNAs [20]. For example, an encoded protein (a putative polypeptide of 124 aa) and a linked miRNA (miR-21) can be potentially expressed coordinately from the same sequence region to generate primary miRNA [21]. Thus, after HCRSV infection in kenaf (*Hibiscus cannabilis* L.) plants, it is plausible that the p23 coding region is able to generate both the p23 protein and vir-miRNAs. A total of five vir-miRNAs have been predicted from the HCRSV genome (Fig. 1) using Vir-Mir database (http://alk.ibms.sinica.edu.tw). In this regard, we investigated if HCRSV RNA is found in the nucleus, where vir-miRNAs are generated.

## Results

### Localization of HCRSV RNA in Nucleus using Fluorescent in Situ Hybridization (FISH) and Highly Purified Nuclei

**Figure 2:**
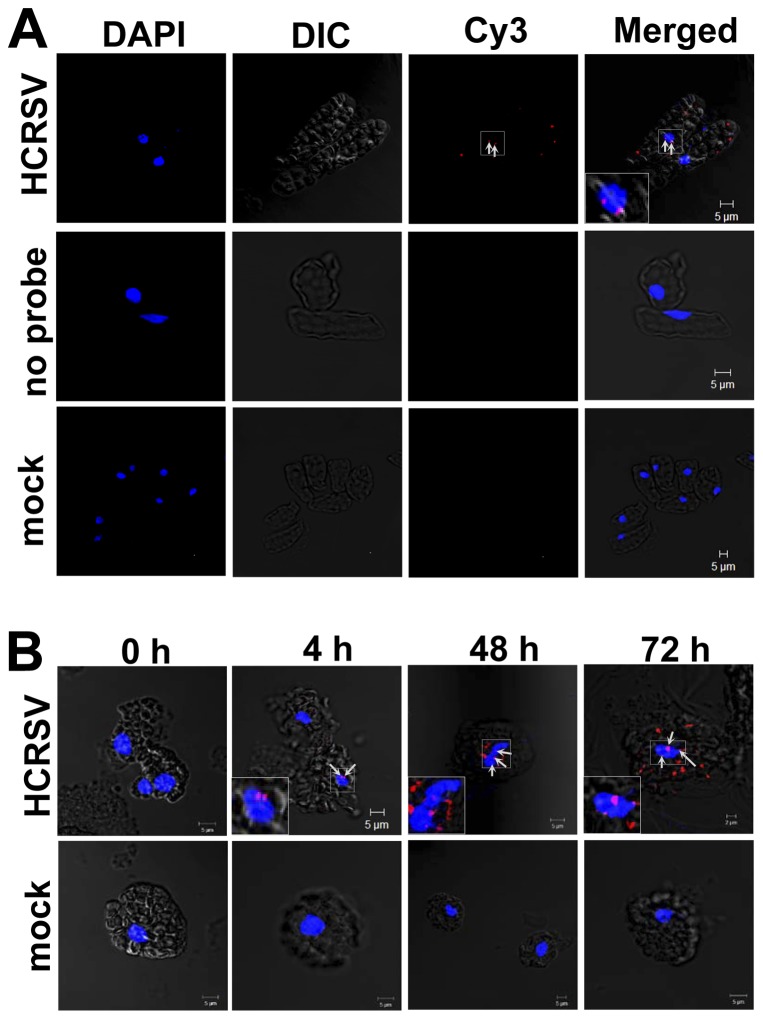
Detection of HCRSV RNA in the nucleus of pre-fixed kenaf cells and isolated protoplasts. A single molecule fluorescent *in situ* hybridization (FISH) method was used, a Cy3-labeled cDNA probe (corresponding to the HCRSV p23 coding region at nt 350-301). DAPI stained nuclei (blue-color foci) were superimposed with the differential interference contrast (DIC) to form a merged image. (*A*) Single cells isolated from mock and HCRSV-infected kenaf leaves were pre-fixed with 4% paraformaldehyde. Cy3 signals were not detected in HCRSV-infected cells without Cy3 probe hybridization and mock inoculated leaf cells (*first* and *second* rows). Single RNA molecules (red dots pointed out by white arrows) were detected in the nuclei of HCRSV-infected cells (red dots within the blue-color foci were enlarged and shown in the insert). (*B*) Kenaf protoplasts were fixed with 4% paraformaldehyde. Cy3 signals were not detected in mock transfected protoplasts. Single RNA molecules (red dots pointed out by white arrows) were detected in nuclei of protoplasts transfected with in vitro transcript of full-length cDNA clone of HCRSV at 0 h, 4 h, 48 h and 72 h, respectively (red dots within the blue-color foci were enlarged and shown in the insert).

Strong Cy3 signals (red dots) were detected in the cytoplasm and nucleus of HCRSV-infected cells hybridized with Cy3-labeled probe (corresponding to the HCRSV p23 coding region at nt 350-301). The specific Cy3 red dots present in the nucleus were highlighted with white arrows (Fig. 2A, first row). No signal was detected from two negative controls, HCRSV-infected cells without the probe and mock inoculated cells hybridized with the probe (Fig. 2A, second and third rows).

Considering HCRSV RNA was detected in the nucleus, we examined when did the RNA enter the nucleus? This was analyzed by a time-point study using isolated kenaf protoplasts transfected with HCRSV *in vitro* transcripts and the protoplasts were collected at 0, 2, 4, 17, 24, 36, 48 and 72 h post transfection (hpt). The results showed that as early as 4 hpt, HCRSV RNA was detected in the nucleus of the transfected protoplasts. Time points at 0, 4, 48 and 72 hpt were selected to show the final merged images. The intensity of the red dots observed represent an increase in the amount of HCRSV RNA at 48 and 72 hpt, indicating that replication of HCRSV RNA increased gradually (Fig. 2B). In order to further confirm the Cy3 signals were co-localized with DAPI (diamidino-2-phenylindole) stained nuclei, three-dimensional images were shown (Movie S1, S2, S3, S4). As expected, there was no signal detected in negative control mock transfected protoplasts (Fig. 2B). This is the first report of a viral RNA genome to be detected in the nucleus through visualization of the red dots.

**Figure 3:**
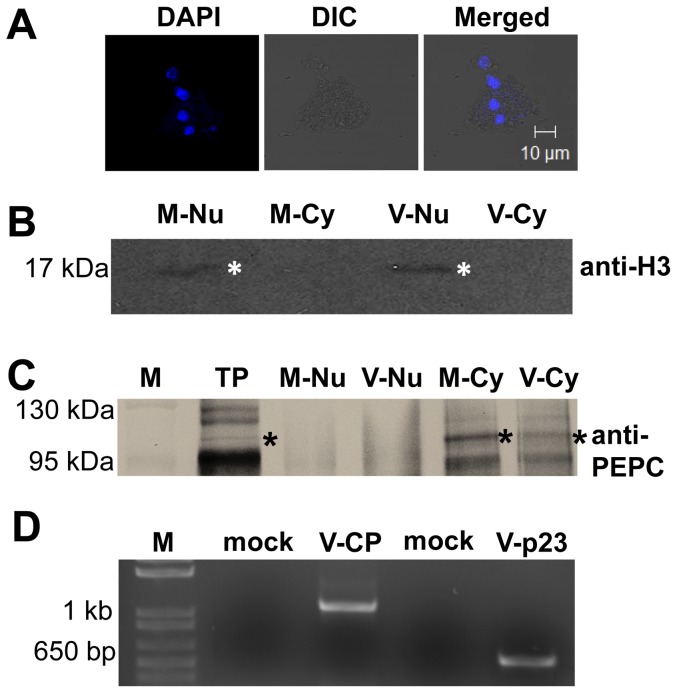
HCRSV RNA was detected in total RNA from purified nuclei. (*A*) Nuclei purified from mock and HCRSV-infected kenaf leaves using CelLytica™ Plant Nuclei Isolation/Extraction Kit. Nuclei were stained with DAPI (Bar = 10 µm). (*B, C*) Verification of the purity of nuclear and cytoplasmic fractions using histone 3 (H3) and phosphoenolpyruvate carboxylase (PEPC) antibodies. The same amount of proteins from each fraction was analyzed by western blots to show the specific localization of nuclear H3 and cytoplasmic PEPC. Total protein, TP. Nuclear and cytoplasmic fractions from mock and HCRSV-infected leaves are represented by (M-Nu and M-Cy) and (V-Nu and V-Cy), respectively. (*D*) P23 and CP genes were amplified from total RNA extracted from HCRSV-infected kenaf nucleus. Mock represent total RNA from buffer inoculated kenaf leaves; V represents total RNA from HCRSV-infected kenaf leaves. The size for HCRSV p23 and CP genes were 630 bp and 1140 bp, respectively.

In order to further demonstrate the presence of HCRSV RNA in the nucleus, another approach was undertaken. Highly purified kenaf nuclei were isolated from HCRSV-infected and mock inoculated kenaf plant leaves. The individual nucleus was investigated by confocal microscopy with DAPI staining (Fig. 3A). Nuclear and cytoplasmic markers, histone 3 (H3) and phosphoenolpyruvate (PEPC), respectively, were used for immunoblot assays to verify the purity of the isolated nuclei. The H3 (∼17 kDa) and PEPC (∼100 kDa) were detected only in their respective nuclear and/or cytoplasmic fractions of mock and HCRSV-infected kenaf leaves (Fig. 3B, 3C). Using the Western blot verified high purity nuclei, total RNA was extracted. RT-PCR results showed that both the p23 (located at the 5′ end of viral genome) and CP genes (located at the 3′end of viral genome) were amplified from total RNA of the HCRSV-infected kenaf nuclei. As expected, no band was amplified from mock plant nuclei (Fig. 3D). This approach further confirmed that HCRSV RNA was present in the nuclei of HCRSV-infected kenaf cells.

### A Novel NLS was Detected in the P23

**Figure 4:**
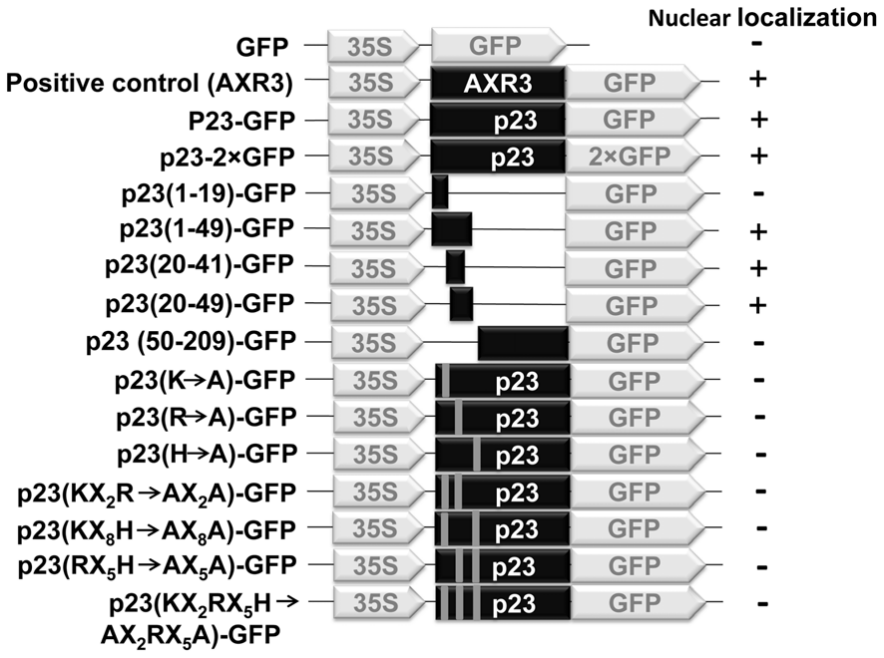
Schematic presentation of constructs of HCRSV p23 and its deletion mutants fused with GFP. 35S represents cauliflower mosaic virus transcription promoter. Black color rectangles represent the p23 and its partial deletion mutants of HCRSV. GFP represents green florescent protein. The grey bars located within the p23 open reading frame represent the mutated amino acid(s).

**Figure 5:**
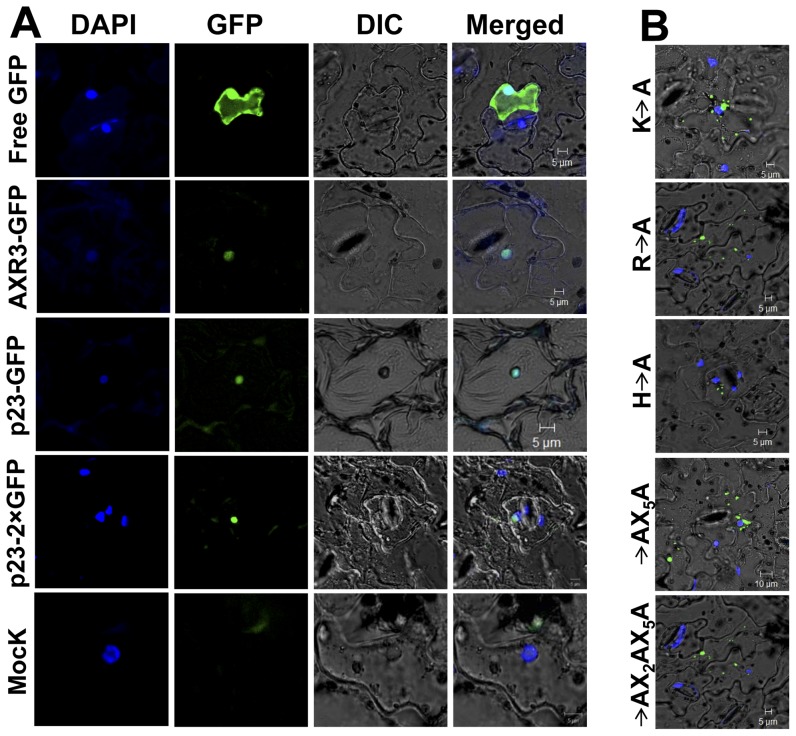
Nuclear localization of the p23 protein of HCRSV. DAPI-stained nuclei (blue-color foci) were superimposed onto the differential interference contrast (DIC) image to form a merged image. (*A*) Kenaf leaves were infiltrated with free GFP and the signal was present in the entire cell including nucleus; p23-GFP and p23-2×GFP fusion proteins were only detected in the nucleus, similar to the positive control AXR3-GFP fusion protein. Free GFP represents agro-infiltration with vector lack of inserted gene. Mock represented agro-infiltration and no GFP signal was detected. (*B*) Localization of p23-GFP fusion protein to nucleus was affected by basic amino acid(s). Among the three basic amino acids (H, R and K) of p23 (20–41)-GFP protein, mutation in any one of the basic amino acids abolished nuclear localization of the p23-GFP fusion protein. Representative mutant images (H→A, R→A, K→A, RX_5_H→AX_5_A, KX_2_RX_5_H→AX_2_AX_5_A) were shown.

After showing the HCRSV RNA was present in the nucleus, the next question is how does the viral RNA enter the nucleus? It has been reported that a nuclear localization signal (NLS) can facilitate RNA to enter nucleus [7]. We asked if there is an NLS existed in any viral protein of HCRSV? We found that an open reading frame (ORF) p23 of HCRSV is predicted to be a transcription factor and it contains a DNA binding motif. Since transcription factors are localized in the nucleus, the first step was to test the localization of the p23. Agro-infiltration transient expression experiments were performed in order to study the p23 sub-cellular localization. The schematic presentations of the p23 fusion protein constructs were shown (Fig. 4). Results showed that strong signal was detected in the entire cell including nucleus when kenaf leaves were infiltrated by a free GFP (Agro-35SpGreen) (Fig. 5A, first row). Similar to a positive control Agro-35SpGreen+AXR3-GFP, kenaf leaves infiltrated with Agro-35SpGreen+p23-GFP, GFP signal was only present in the nucleus (Fig. 5A, second and third rows). In order to exclude the possibility that p23-GFP is small enough to freely diffuse into nucleus, a construct 35SpGreen+p23-2×GFP tagged with two GFPs was generated. The results showed that signal from 2×GFP was also detected mainly in the nucleus; further confirming that the p23 was localized in the nucleus (Fig. 5A, fourth row). No GFP signal was detected in the mock infiltrated leaves (Fig. 5A, last row).

**Figure 6:**
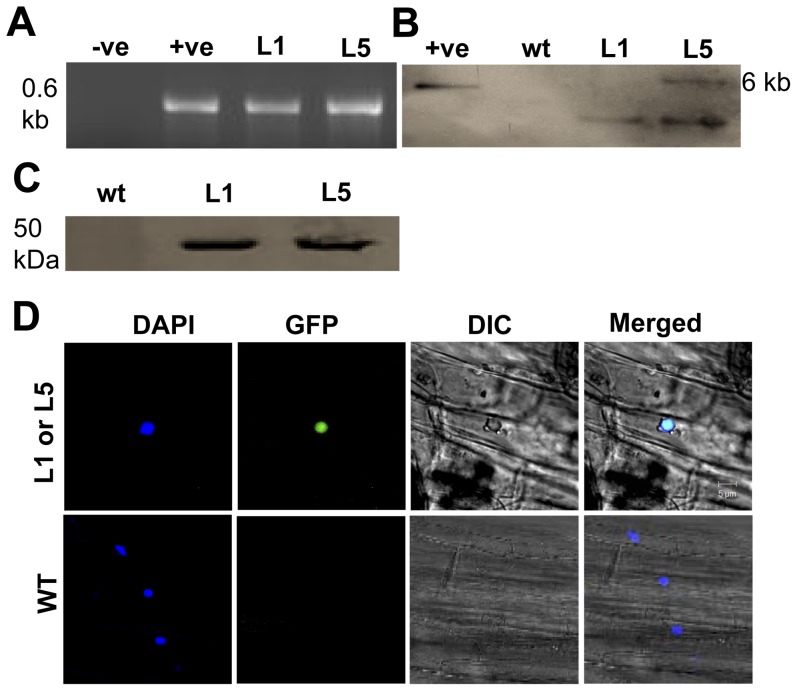
Localization of p23 of HCRSV in nucleus of transgenic *Arabidopsis*. (*A*) Detection of p23 gene transcript in transgenic *Arabidopsis* using vector and the p23 gene-specific primers in RT-PCR. (*B*) The p23 was detected in transgenic lines L1 and L5, respectively. Southern blot showed that the p23 was successfully incorporated into transgenic *Arabidopsis*. L1 and L5 showed one and two copies, respectively. (*C*) Western blot for p23-GFP fusion protein detection in putative transgenic plants, anti-GFP antibody was used for immune-blot. (*D*) DAPI stained nuclei (blue-color foci) were superimposed onto the differential interference contrast (DIC) image, forming a merged image. The p23-GFP fusion protein was detected in the nucleus of p23-GFP transgenic *Arabidopsis* plants. +ve, positive control; −ve, negative control; wt, Wild type; L1 and L5, putative *Arabidopsis* transgenic p23 lines 1 and 5.

To further prove that the p23 was localized in the nucleus, in addition to the transient expression experiments, stable p23 expression was also tested. To achieve this, a p23-GFP transgenic *Arabidopsis* was generated. Putative p23 transgenic line 1 (L1) and line 5 (L5) were determined by PCR using vector forward primer (35S, GACCCTTCCTCTATATAAGGAAGTTC) and gene reverse primer (p23R, CGCGGATCCCGGGCGAGTACCCCTGAAA) (Fig. 6A), as well as southern blot (Fig. 6B) using DIG-labeled p23 cDNA probe and western blot using anti-GFP antibody (Fig. 6C) to detect if the p23-GFP gene was successfully transformed into wild type *Arabidopsis*. L1 and L5 were used to confirm nuclear localization of p23. Similar to the transient expression results, Prominent GFP signal was detected in the nucleus (Fig. 6D, first row). In contrast, no GFP was detected in the wild type *Arabidopsis* (Fig. 6D, second row).

**Figure 7:**
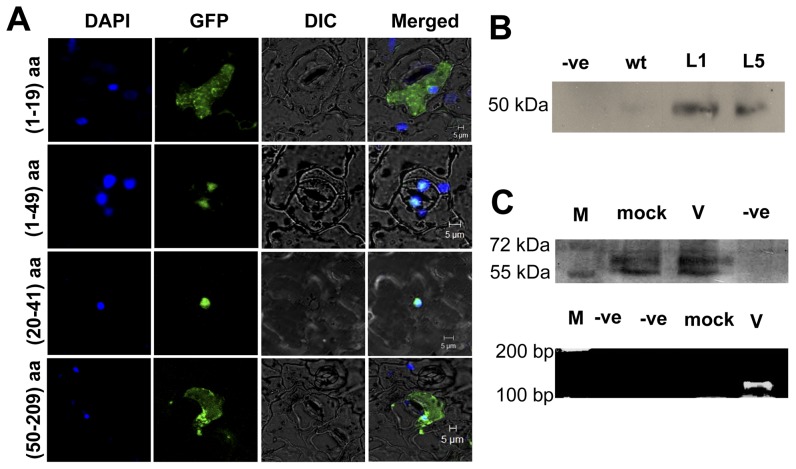
Detection of nuclear localization signal (NLS) in p23 of HCRSV. (*A*) DAPI stained nuclei (blue-color foci) were superimposed onto the differential interference contrast (DIC) image to form a merged image. The fragments of GFP fusion proteins pGreen+p23 (1–19 aa)-GFP and pGreen+p23 (50–209 aa)-GFP, which did not contain NLS, were present in the entire cell. The pGreen+p23 (1–49 aa), p23 and p23 (20–41 aa)-GFP, both encompass NLS fragment. GFP signal was mainly present in the nucleus. (*B*) Wild type (wt) and p23-GFP transgenic *Arabidopsis* L1 and L5 lines were used as plant materials; anti-GFP antibody was used to carry out co-immunoprecipitation. The pull down protein was probed with anti-importin α antibody. Importin α was detected in the CO-IP samples. (*C*) Mock and HCRSV-infected kenaf leaves were used for RNA-chromatin immunoprecipitation (CHIP). Before immmunoprecipitation, importin α was detected in sonicated mock and HCRSV-infected kenaf leaves. Truncated p23 (46–183 aa) of HCRSV was detected only in HCRSV-infected leaves using RT-PCR from eluted RNA. M, protein markers.

Even if the p23 can be detected in the nucleus, it is not essential for it to contain a NLS since it can bind to other carrier protein to enter nucleus. Hence, the p23 was divided into different fragments, according to its predicted cleavage sites, corresponding constructs were shown (Fig. 4). Firstly, HCRSV p23 fragments (1–49 aa) and p23 (50–209 aa) were constructed into pGreen-GFP vector. Only the (1–49 aa) fragment was localized in the nucleus. The p23 (1–49 aa) fragment was further cut into two parts - (1–19 aa) and (20–49 aa). The latter fragment was present in the nucleus. Subsequently, eight aa from the C-terminal of p23 (20–49 aa) were removed, leaving only the putative DNA binding motif (20–41 aa). Finally, the p23 (20–41 aa) fragment was determined as the shortest fragment that was localized in the nucleus. Representative images of nuclear localization were shown (Fig. 7A). Fragment containing p23 (20–41 aa), GFP signal was largely present in the nucleus (Fig. 7A, second and third rows). However, for fragments p23 (1–19 aa) and (50–209 aa), GFP signal was present in the entire cell (Fig. 7A, first and last rows).

In order to confirm the novel NLS in the p23, mutational analysis was performed. Several aa mutations were made in the fragment of p23 (20–41 aa) and localization of fusion protein was investigated. From the mutant clones, three basic aa mutations (H, R and K) located in positions (20–41 aa) in the p23 of HCRSV. Any one of the basic aa mutation abolished nuclear localization of p23-GFP fusion protein. A total of seven mutants were generated by replacing each basic aa with an alanine residue (H→A, R→A, K→A, KX_2_R→AX_2_A, RX_5_K→AX_5_A, HX_8_K→AX_8_A and KX_2_RX_5_H→AX_2_AX_5_A). Six representatives were shown (Fig. 5B). Compared with the localization of p23-GFP (Fig. 5A, third row), mutant fusion proteins nuclear localization was abolished (Fig. 5B). However, mutants with different leucine residues mutated to alanine residue in the fragment of p23 (20–41 aa) retained its nuclear localization.

After proving the presence of an NLS in the p23, its mode of entry into nucleus was investigated. It has been reported that importin proteins can help other proteins with NLS to enter nucleus [7]. Transgenic *Arabidopsis* expressing p23-GFP and anti-GFP antibody were used to perform co-immunoprecipitation (co-IP). Eluted protein was probed with anti-importin α antibody. Compared with the wild type plant, the importin α was only detected in the protein extract from p23-GFP transgenic *Arabidopsis* plants (Fig. 7B), indicating that the p23-GFP enters nucleus with the aid of importin α.

Our hypothesis is that importin α, p23 and HCRSV RNA form a complex to enter nucleus. For detecting if importin α can bind HCRSV RNA and facilitates its entry into nucleus, RNA-chromatin immunoprecipitation (RNA-CHIP) experiment was carried out. The results showed that importin α was detected in both sonicated mock and HCRSV-infected kenaf leaves, suggesting that importin α was present in the cross-linked protein-RNA complex (Fig. 7C, first row). For RNA that was eluted from the agarose beads, the p23 gene encoding fragment (46–183 nt) was detected only in HCRSV-infected kenaf leaves, but not in mock inoculated leaves (Fig. 7C, second row). The results indicated that the NLS of p23 facilitates the entry of HCRSV RNA into nucleus through its binding to impotin α.

### Prediction and Detection of vir-miRNA In Total RNA Extracted from Highly Purified Kenaf Nuclei of HCRSV-Infected and Agro-Infiltrated Leaves

After showing how HCRSV RNA could enter nucleus, the next question is why it enters? miRNA was studied extensively in recent years and many viral-miRNAs have been discovered. In addition, according to the vir-miRNA prediction database (Vir-Mir), five candidates (Fig. 1A, 1B), namely hcrsv-miR-H1-5p (nt 305-394), hcrsv-miR-H2-5p (nt 537-622), hcrsv-miR-H3 (nt 2096-2180), hcrsv-miR-H4-3p (nt 2836-2910) and hcrsv-miR-H5-3p (nt 3247-3337), respectively, were predicted from the complete nucleotide sequence of HCRSV (NC_003608).

**Figure 8:**
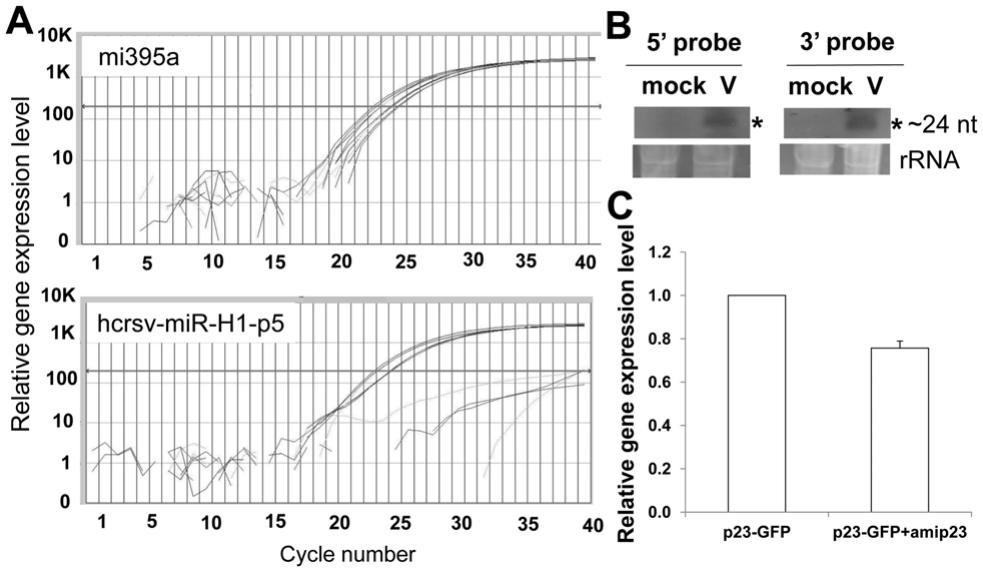
Detection of HCRSV vir-miRNA. (*A*) Vir-miRNA hcrsv-miR-H1-p5 was detected using Taqman real time PCR. miR395a was used as a positive control; hcrsv-miR-H1-p5 was detected from total RNA extracted from nuclei of HCRSV-infected leaves. No RNA template was used as a negative control. (*B*) Vir-miRNA hcrsv-miR-H1-p5 was detected using northern blot. The amount of total RNA mock and HCRSV-infected samples used was 15 µg each. Small RNAs were detected from total RNA extracted from nuclei of HCRSV-infected kenaf leaves using both 5′- and 3′-DIG-end-labeled miRNA probes (Hcmi305pro-5p, HCRSV nt 344-319; Hcmi305pro-3p, HCRSV nt 382-357). (*C*) HCRSV p23 was downregulated by vir-miRNA. One of the candidates vir-miRNA (hcrsv-miR-H1-5p) was constructed into an artificial miRNA expression vector (amip23). Kenaf plant leaves were infiltrated with pGreen+p23-GFP or mixture of pGreen+p23-GFP and vir-miRNA hcrsv-miR-H1-p5.

In order to prove that there is vir-miRNAs existed in the HCRSV, two approaches were carried out. Firstly, mature miRNAs were quantified using TaqMan® MicroRNA Assays, in which the stem-loop RT primers are more specific than the conventional ones. The results showed that vir-miRNAs were detected in total RNA extracted from HCRSV-infected kenaf leaves. In order to exclude the involvement of siRNAs, total RNAs isolated from highly purified kenaf nuclei were also used for the vir-miRNA detection, using TaqMan real-time PCR. A plant conserved miRNA mi395a (as a positive control) showed high expression level (Fig. 8A, upper panel), indicating the feasibility of TaqMan real time PCR system. Similar to the positive control, strong vir-miRNA (hcrsv-miR-H1-p5) signal was detected from total RNA of isolated purified nuclei. Secondly, another approach was to use northern blot to verify the existence of vir-miRNAs. The results showed that mature miRNAs (∼24 nt) were detected using both 5′- and 3′-DIG-labeled 26 nt miRNA probes in northern blot analysis (Fig. 8B). Since the total RNA was extracted from highly purified nuclei, siRNAs were excluded. In addition, the siRNAs are not present in nucleus as they are produced in the cytoplasm. Furthermore, these small RNAs, which were detected from TaqMan real-time PCR (commercial designed primers specific for miRNAs) and northern blot, were not random degradation fragments of HCRSV, because these small RNAs were detected from at least 3 independent experiments.

In order to further prove that the small RNA signals detected were miRNAs, but not siRNAs that were generated from HCRSV infection. Experiments using *Agrobacterium*-infiltrated p23-GFP gene was performed. Highly purified nuclei were extracted from corresponding genes (GFP, p23-GFP and p27-GFP) infiltrated into kenaf leaves after 72 h. Then total RNAs were extracted from these purified nuclei. RT-PCR results showed that the sequence encoding p23 was only detected from p23-GFP *Agrobacterium*-infiltrated kenaf leaves, but not from empty vector (GFP) or negative control (p27-GFP). Similarly, vir-miRNA hcrsv-miR-H1-5p was also detected only in total RNA from the nuclei of p23-GFP agro-infiltrated kenaf leaves, using TaqMan real time PCR and northern blot.

## Discussion

In this study, we have identified the presence of a viral RNA genome in nucleus. Using FISH, we demonstrated the presence of the Cy3-labelled probe targeting viral RNA in the nucleus of single cells isolated from paraformaldehyde-fixed HCRSV-infected kenaf leaves and protoplasts. Western blot analysis verified that the isolated nuclei were highly purified. Using total RNA extracted from the purified nuclei, both of the p23 coding region (located near the 5′-end of the viral genome) and the CP gene (located near the 3′-end of the viral genome) were amplified using RT-PCR. Finally, using the customized stem loop primers, vir-miRNA was detected using Taqman® real-time PCR. In addition, small RNAs were detected using both 5′ and 3′ miRNA probes, suggesting that both of the mature miRNA strands may have higher expression level in the cells [22]. In theory, since vir-miRNAs are generated in the nucleus in plants, nucleated and enucleated protoplasts appeared to be a good method to compare the presence or absence of vir-miRNAs in the nucleus. However, vir-miRNAs that are generated in the nucleus are not distinguishable from siRNAs that are generated in the cytoplasm upon virus infection. Both mature vir-miRNAs and siRNAs contain single-stranded RNA molecules of approximately 21–25 nucleotides in length and their sequences are both derived from the same virus genome. As a result, the vir-miRNAs and siRNAs produced separately in the nucleus and in the cytoplasm are indistinguishable. Therefore, isolating highly purified nuclei from HCRSV-infected kenaf leaves and detecting the vir-miRNAs is the only viable method to separate vir-miRNAs from siRNAs.

### Vir-miRNA Hcrsv-Mir-H1-5p Targets the p23 Gene of HCRSV

HCRSV RNA entering nucleus may be used to synthesize vir-miRNAs. Since the first vir-miRNA was discovered, its functions have been studied. It represses both host cellular genes and viral genes to aid viral replication and to function as orthologs of cellular miRNAs. While vir-miRNAs have been discoverred progressively from DNA viruses, there is none reported in RNA viruses. The current knowledge shows that RNA virus replication is associated with the cytoplasmic membranes in the host cells. As the dicer-like processing complex is located in the nucleus and viral RNA is not known to enter nucleus where vir-miRNA is generated, it is not apparent how RNA viruses can be processed to yield mature miRNAs in the cytoplasm [23]. However, a known miR-124, which was artificially inserted into an intron of an nuclear export protein transcript, could be delivered into the cytoplasm of murine fibroblasts by a modified Influenza virus [24]. Since both vir-miRNAs and cellular miRNAs are found in the RNA-induced silencing complex [23], [25], it is deduced that vir-miRNAs can also target viral and cellular mRNAs. One possibility is that vir-miRNAs can target viral RNAs as they are derived from the same genomic sequence. Expression of viral genes, such as that from EBV, Simian virus 40 and Herpes simplex virus, can be down-regulated by their respective vir-miRNAs [26]–[28]. Furthermore, if one or two bases of the vir-miRNAs are changed, its specificity can also be altered. Consequently, the genomic sequences encoding the vir-miRNAs would exhibit rapid evolutionary drift. Thus, co-evolution of vir-miRNAs and their corresponding viral RNA are expected. The second possibility is that vir-miRNAs can target cellular genes. For example, expression of LMP1 protein is modulated by EBV-encoded BamHI-A rightward transcript Cluster 1 miRNAs which targets the 3′ UTR of the *LMP1* gene [28]. Another example is protein thrombospondin 1, which is down regulated by Kaposi sarcoma-associated herpesvirus-encoded miRNAs [29].

Using constructed vir-miRNA hcrsv-miR-H1-5p (amip23) for co-infiltration experiments, we tested if hcrsv-miR-H1-5p can target the p23 to regulate its expression. Our results showed that amip23 can target and downregulate p23 gene in co-infiltrated pGreen-p23-GFP/amip23, as compared with p23-GFP infiltrated alone, or pGreen-p23-GFP/amip27 (Fig. 8C). As expected, for negative controls pGreen-GFP/amip23, pGreen-CP-GFP/amip23, there was no p23 gene expression detected. From our previous findings, p23 is essential for HCRSV replication and any mutations introduced into the ORF of p23 would abolish viral replication in kenaf plants [5]. Therefore, the presence of hcrsv-miR-H1-5p may inhibit viral replication. As a result, replication of the virus is regulated. In addition, the p23 ORF sequence, from which hcrsv-miR-H1-5p is derived, might also alleviate evolutionary constraints. It is also possible that hcrsv-miR-H1-5p will target host genes which are involed in plant defence processes. Negative regulation of host defence genes will allow higher virus replication.

### The NLS of p23 Facilitates Importin α and HCRSV RNA to Enter Nucleus

Our results showed that the p23 of HCRSV contains a novel NLS, which is different from the classical NLS (cNLS) for nuclear protein import. The best characterized transport cNLS consists of either one or two clusters of basic aa. One type of the cNLSs, as represented by P**KKKRR**V, is from the SV40 large T antigen [30]–[33]. The other type, as represented by **KR**PAATKKAGQA**KKKK** , is from the nucleoplasmin [34]. The rules are that for the single motif, it is characterized by a cluster of 5 basic aa residues and the double motifs consist of two clusters of basic aa residues separated by 9–12 aa residues. However, not all experimentally known NLSs follow the same rules [35]–[42]. Furthermore, many non-nuclear proteins also contain such clusters of basic aa residues. Similar to the non-cNLSs, the verified NLS of p23 (LSPQLLKLSRTPVSLHEILASL) which is leucine rich and highly hydrophobic. The NLS of p23 overlaps with the predicted DNA binding domain. It agrees with a previous report in which the authors found that there is an overlap in the NLS and DNA binding region of 90% of the proteins for which both the NLS motif and DNA-binding regions were found [6]. It is reported that positively charged residues are abundant in NLSs and some of them can bind to importins to aid nuclear proteins to enter nucleus [36]. Our results also showed that mutation to any of the 3 positively charged basic aa - arginine (R), lysine (K) and histidine (H), would disrupt p23 nuclear import.

For a protein with an NLS entering nucleus, the molecular basis for recognition of a cNLS by importin α has been defined using x-ray crystallography [43]–[46]. Since the cNLS is oftern thought of as the typical NLS, many proteins use the classcial import pathway to enter nucleus. Our co-IP results showed that importin can bind to the p23, indicating that it also adopt the classical way for the entry of nucleus. The reason we used the p23-GFP transgenic *Arabidopsis* as co-IP materials is due to low expression level of p23 *in vivo*. The p23 is predicted to be a transcription factor. In addition, since the p23 is highly hydrophobic, it is difficult to express it. Attempts to produce a monoclonal antibody to p23 was unsuccessful. Using the p23 with a GFP tag (p23-GFP) which was driven by a strong Cauliflower mosic virus (CaMV) 35S promoter, it is possible to obtain sufficient quantity of p23 protein for immunoprecipitation. Furthermore, RNA-CHIP experiments also showed that importin α can bind to HCRSV RNA, forming a p23-importin α-HCRSV RNA complex, and facilitates HCRSV RNA to enter nucleus.

**Figure 9:**
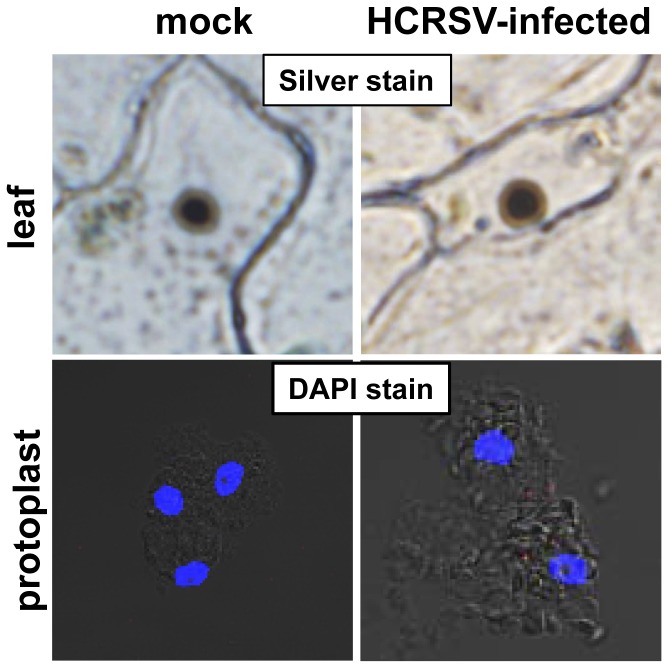
Nucleolus of mock and HCRSV infected kenaf leaf and protoplasts. Upper panel shows silver staining of nucleolus (dark brown color) in a kenaf epidermal leaf cell. Lower panel shows DAPI stained nucleoli (dark spots in the blue nucleus) in 2–3 kenaf protoplasts.

Viral genetic material enterring nucleus can affect nucleus, for example, the target of rapamycin (TOR) protein, which is a conserved regulator of ribosome biogenesis, can modulate nucleolar structure [47]. The CaMV ORF VI product (P6) is an essential determinant for the formation of viroplasms [48]. Cucumber mosaic virus 2b protein nucleolar localization was affected by its respective mutants [49]. However, from our silver and DAPI staining of nucleus, there were no differences in nucleolus between mock and HCRSV-infected kenaf cells (Fig. 9).

### The Presence of Viral RNA in the Nucleus May Unravel Novel Funcitons in Gene Regulation

Our results showed that similar to DNA viruses, viral RNA genome can also enter nucleus. This will open up many studies to unravel more functions of RNA viruses. For example, do all RNA viruses enter nucleus of host cells? Does viral RNA interact with host components in nucleus to regulate gene expression? Better understanding on how viral replication is regulated will allow researchers to control viral diseases by designing specific molecules to target viral pathogens in future.

In conclusion, we have identified the presence of a (+)-sense ss viral RNA in nucleus, where vir-miRNAs are generated. In addition, a novel NLS in p23 facilitates viral RNA to enter nucleus through its binding to importin α.

## Materials and Methods

### Plant Materials, Plasmid Construction and Generation of Transgenic *Arabidopsis*

Kenaf seedlings were grown under 16 h light 8 h dark cycles at 25°C. The HCRSV p23 coding region was PCR amplified using p23-F: CCGGAATTCATGCTTTCTCAATTGCTTTC and p23-R: CGCGGATCCCGGGCGAGTACCCCTGAAA primers. A construct 35SpGreen-p23-GFP was transferred into *Agrobacterium*. Cloning of p23 with predicted cleavage fragments were constructed as p23 (1–49 aa), p23 (50–209 aa), p23 (1–19 aa), p23 (20–49 aa) and p23 (20–41 aa) were made to identify the NLS. Within the p23 (20–41 aa) fragment, mutations at three basic aa (K, R and H) were constructed using routine high-fidelity PCR (KAPA) by point mutations. In addition, three leucine residues (as negative controls) were mutated to confirm the NLS in the p23. *Arabidopsis* seedlings were grown in long-day conditions (16 h light/8 h dark, 23°C). The p23-GFP gene was transformed into wild type *Arabidopsis* using floral dip method following published protocols [50].

### Verification of Putative Transgenic *Arabidopsis* Plants using Southern Blot

Southern blot was performed strictly following a protocol (Roche, DIG application manual for filter hybridization). Briefly, Ten µg genomic DNA was digested with EcoRI and separated on an agarose gel overnight. After denaturing with buffer (0.5M NaOH, 1.5 M NaCl), the DNA was transferred to nylon membrane using capillary transfer method. The transferred DNA was cross linked by UV and hybridized with DIG-labeled p23 DNA probe at 42°C, 16 h. Finally, the membrane was probed with anti-DIG antibody before it was developed with CPSD substrate.

### Construction of Artificial vir-miRNA Hcrsv-miR-H1-5p

Vir-miRNA hcrsv-miR-H1-5p was engineered into the endogenous miR319a precursor (plasmid pRS300) by site-directed mutagenesis, following the protocol by Rebecca Scheab Max-Plank Institute for Developmental Biology, Tuebingen, Germany (2005) (Ossowski Stephan, Fitz Joffrey, Schwab Rebecca, Riester Markus and Weigel Detlef, personal communication). The artificial miRNAs amip23 and amip27 (constructed vrial miRNAs which can target p23 (375-394 nt) and p27 (2622-2614 nt), respectively) were generated.

### *Agrobacterium tumefaciens*–Mediated Transient Expression

*Agrobacteria* culture grown to OD_600 nm_ = 1.0–1.5 was harvested, containing the pGreen-p23-GFP, truncated p23 fragments or artificial vir-miRNA plasmids. The cell pellet was resuspended in buffer (pH = 7) containing 10 mM each of MgCl_2_ and MES, and 100 µM acetosyringone, infiltration was performed after 4 h. Negative controls include original plasmid (35S pGreen+GFP) and 35S pGreen+p27+GFP. All the infiltration transient expression experiments were repeated twice. For co-infiltration experiments, agroinfiltration mixtures of pGreen-GFP/amip23, pGreen-p23-GFP/amip23, pGreen-CP-GFP/amip23, pGreen-GFP/amip27, pGreen-p23-GFP/amip27 were prepared.

### Co-immunoprecipitation Assay

*In vivo* co-IP was carried out in accordance with the manufacturer's protocol (Pierce, Rockford, IL, U.S.A.). Briefly, 7 days later, wild type and p23-GFP transgenic *Arabidopsis* seedlings (0.1 g) were homogenized on ice in 0.2 ml of protein extraction buffer [220 mM Tris–HCl, pH 7.4, 250 mM sucrose, 50 mM KCl, 1 mM MgCl_2_, 2 mM phenylmethylsulfonyl fluoride, 10 mM β-mercaptoethanol, and 1× complete EDTA-free protease inhibitor (Sigma, St. Louis)]. The extract was centrifuged at 12,000× *g* for 10 min to remove cellular debris, and the supernatant (100 µl aliquots) was mixed with equal volumes of coupling buffer and incubated for 2 h with amine-reactive gel previously coupled to 750 µg rabbit anti-GFP antibody. The amine-reactive gel was washed three times with coupling buffer and the immunoprecipitated proteins were subsequently eluted from the gel with elution buffer. The immunoprecipitates were probed with anti-importin α antibody.

### RNA-CHIP Analysis

CHIP experiment was performed following previous protocol [51]. Mock and HCRSV- infected kenaf leaves were used for cross-link with formaldehyde. Importin α was used for precipitation. Slight modifications were made for RNA-CHIP analysis. RNAnase inhibitor was added before sonication. For RNA extraction from eluent, NaCl was added to a final concentration of 200 mM and placed at 65°C for at least 2 h for reverse crosslinking. Next, final concentration 40 mM Tris-Cl (pH 6.5), 10 mM EDTA, and 40 µg/ml of proteinase K was added to each sample and incubated at 42°C for 45 min. Samples were subjected to phenol∶chloroform purification and isopropanal precipitation. RNA pellets were washed once in nuclease-free 75% ethanol, air-dried briefly, and resuspended in 20 µl of nuclease-free water. DNA from the samples was removed by using of DNAse I. RNAs can be reverse transcribed using Superscript III RT (Invitrogen) and performed PCR. The primers used were listed in Table S1.

### Preparation of Plant Cells and Protoplasts for Fluorescent in situ Hybridization (FISH) and Silver/DAPI Staining

Mock and HCRSV-infected kenaf leaves were cut into 1 mm×1 mm and fixed with 4% (w/v) paraformaldehyde, 2.5% (w/v) sucrose in 0.1 M phosphate buffer (pH 7.2), 2 h at room temperature. The cells were washed twice with sterile H_2_O before digested with filter-sterile cellulase (0.8%) and macerase (0.25%) for 16 h. The cells were isolated accordingly [52]. Protoplasts were transfected with *in vitro* transcribed RNA from full-length HCRSV cDNA (Ambion, mMESSAGE). After 0, 4, 48 and 72 h transfection, protoplasts were collected and fixed with fixation buffer described above. The FISH procedures [53] were adopted with slight modifications. Fifty ng Cy3-labeled HCRSV cDNA probe (5′-GTTGGAGTGCCCCCAAAATGTAGCTTTGCTGCGTTGCCGCATGGAGAGC-3′) for each reaction was used to localize HCRSV RNA. Images were obtained using a confocal laser scanning microscope (Carl Zeiss LSM510 META confocal, Germany).

Lower epidermal layer of kenaf leaves was peeled off using a pair of forceps and stained with DAPI or silver according to the protocol ((http://www.le.ac.uk/bl/phh4/agnor.htm) described by Heslop-Harrison and Schwarzacher of the Molecular Cytogenetics Research Group at University of Leicaster, U.K.

### Isolation and Verification of Highly Purified Kenaf Nuclei and detection of HCRSV RNA

Instructions of the CelLytic PN Plant Nuclei Isolation/Extraction Kit (Sigma-Aldrich) were strictly followed. Blotted proteins were probed with anti-phosphoenolpyruvate carboxylase (PEPC) and anti-histone 3 (H3), followed by alkaline phosphatase-conjugated protein A or anti-rabbit IgG peroxidase conjugate. Finally, membranes were visualized using nitroblue tetrazolium/5-bromo-4-chloroindol-3-yl phosphate staining or Thermo Scientific SuperSignal West Pico Chemiluminescent substrate. RT-PCR primers designed to amplify the p23 and the CP coding regions of HCRSV were carried out, using total RNA extracted from the highly purified kenaf nuclei.

### Preparation of Total RNA, Reverse Transcriptase and real-time PCR

Total RNAs were extracted using TRIzol reagent (Invitrogen). Reverse transcription was carried out using reverse transcriptase (Applied Biosystems, USA,). HCRSV CP gene was amplified with H-CP-F and H-CP-R (Table S1).

### Prediction and Detection of vir-miRNAs

Candidate sequences for vir-miRNA in HCRSV were predicted using the Vir-Mir database (http://alk.ibms.sinica.edu.tw) [54], [55].

Standard TaqMan assays for vir-miRNA were obtained from the custom TaqMan small RNA assay design tool (https://products.appliedbiosystems.com). Since the probe sequence is derived from proprietary bioinformatics analysis by the company, an assay ID CSKAJSI (Applied Biosystems), instead of the actual sequence, was assigned to a predicted HCRSV vir-miRNA for future ordering. The hcrsv-miR-H1-5p was detected subsequently using TaqMan real-time PCR [56].

Two DIG-end-labeled probes (26 nt each) labeled with DIG oligonucleotide tailing kit (Roche Applied Science) were used to detect miRNA. The probes used were:

Hcmi305pro-5p (5′-GTGCCCCCAAAATGTAGCTTTGCTGC-3′) and

Hcmi305pro-3p (5′-GCCGCACAGCAAGCTCATTCGAGCGC-3′).

Northern blot was performed following a protocol [57] with some modifications. Prehybridization was performed 1 h at 65°C after UV crosslinking. Hybridization was performed at room temperature for 16 h. The membrane was washed three times at room temperature with buffer containing 6× SSC and 0.2% SDS and once at 48°C for 10 min.

## Supporting Information

Movie S1 **Three dimensional rotating image of a mock-transfected protoplast 72 hpi using FISH method.** DAPI-stained nuclei (blue-color foci) were superimposed onto the differential interference contrast (DIC) image. No Cy3 signal (red color) was detected in the nuclei of the mock transfected kenaf protoplast.
(MPG)

Movie S2 **Three dimensional rotating image of a HCRSV-transfected protoplast 72 hpi using FISH method.** DAPI-stained nuclei (blue-color foci) were superimposed onto the differential interference contrast (DIC) image. Single RNA molecules (red dots within the blue-color foci) were detected in nuclei of the protoplast transfected with *in vitro* transcript of full-length cDNA clone of HCRSV.
(MPG)

Movie S3 **Three dimensional animation images of a mock-transfected protoplast 72 hpi using FISH method.** DAPI-stained nuclei (blue-color foci) were superimposed onto the differential interference contrast (DIC) image. No Cy3 signal (red color) was detected in the nuclei of the mock transfected kenaf protoplast.
(MPG)

Movie S4 **Three dimensional animation images of a HCRSV-transfected protoplast 72 hpi using FISH method.** DAPI-stained nuclei (blue-color foci) were superimposed onto the differential interference contrast (DIC) image. Single RNA molecules (red dots within the blue-color foci) were detected in nuclei of the protoplast transfected with *in vitro* transcript of full-length cDNA clone of HCRSV.
(MPG)

Table S1 **Primers used in this study.**
(DOC)

## References

[1] Koonin EV, Senkevich TG, Dolja VV (2006) The ancient Virus World and evolution of cells. Biol Direct 1: 29. 16984643 10.1186/1745-6150-1-29 PMC1594570

[2] Ahlquist P (2002) RNA-dependent RNA polymerases, viruses, and RNA silencing. Science 296: 1270–1273. 12016304 10.1126/science.1069132

[3] Chiba S, Kondo H, Tani A, Saisho D, Sakamoto W, et al (2011) Widespread endogenization of genome sequences of non-retroviral RNA viruses into plant genomes. PLoS Pathog 7: e1002146. 21779172 10.1371/journal.ppat.1002146 PMC3136472

[4] Huang M, Koh DC, Weng LJ, Chang ML, Yap YK, et al (2000) Complete nucleotide sequence and genome organization of hibiscus chlorotic ringspot virus, a new member of the genus Carmovirus: evidence for the presence and expression of two novel open reading frames. J Virol 74: 3149–3155. 10708431 10.1128/jvi.74.7.3149-3155.2000 PMC111815

[5] Liang XZ, Lucy AP, Ding SW, Wong SM (2002) The p23 protein of hibiscus chlorotic ringspot virus is indispensable for host-specific replication. J Virol 76: 12312–12319. 12414971 10.1128/JVI.76.23.12312-12319.2002 PMC136886

[6] Cokol M, Nair R, Rost B (2000) Finding nuclear localization signals. EMBO Rep 1: 411–415. 11258480 10.1093/embo-reports/kvd092 PMC1083765

[7] Wu WW, Pante N (2009) The directionality of the nuclear transport of the influenza A genome is driven by selective exposure of nuclear localization sequences on nucleoprotein. Virol J 6: 68. 19490630 10.1186/1743-422X-6-68 PMC2694790

[8] Taliansky ME, Brown JW, Rajamaki ML, Valkonen JP, Kalinina NO (2010) Involvement of the plant nucleolus in virus and viroid infections: parallels with animal pathosystems. Adv Virus Res 77: 119–158. 20951872 10.1016/B978-0-12-385034-8.00005-3 PMC7149663

[9] Bartel DP (2009) MicroRNAs: target recognition and regulatory functions. Cell 136: 215–233. 19167326 10.1016/j.cell.2009.01.002 PMC3794896

[10] Kidner CA, Martienssen RA (2005) The developmental role of microRNA in plants. Curr Opin Plant Biol 8: 38–44. 15653398 10.1016/j.pbi.2004.11.008

[11] Jones-Rhoades MW, Bartel DP, Bartel B (2006) MicroRNAs and their regulatory roles in plants. Annual Review of Plant Biology 57: 19–53. 10.1146/annurev.arplant.57.032905.105218 16669754

[12] Lu YD, Gan QH, Chi XY, Qin S (2008) Roles of microRNA in plant defense and virus offense interaction. Plant Cell Rep 27: 1571–1579. 18626646 10.1007/s00299-008-0584-z

[13] Sullivan CS, Ganem D (2005) MicroRNAs and viral infection. Mol Cell 20: 3–7. 16209940 10.1016/j.molcel.2005.09.012

[14] Pfeffer S, Zavolan M, Grasser FA, Chien M, Russo JJ, et al (2004) Identification of virus-encoded microRNAs. Science 304: 734–736. 15118162 10.1126/science.1096781

[15] Bennasser Y, Le SY, Yeung ML, Jeang KT (2004) HIV-1 encoded candidate micro-RNAs and their cellular targets. Retrovirology 1: 43. 15601472 10.1186/1742-4690-1-43 PMC544590

[16] Sullivan CS, Grundhoff AT, Tevethia S, Pipas JM, Ganem D (2005) SV40-encoded microRNAs regulate viral gene expression and reduce susceptibility to cytotoxic T cells. Nature 435: 682–686. 15931223 10.1038/nature03576

[17] Cai X, Lu S, Zhang Z, Gonzalez CM, Damania B, et al (2005) Kaposi's sarcoma-associated herpesvirus expresses an array of viral microRNAs in latently infected cells. Proc Natl Acad Sci U S A 102: 5570–5575. 15800047 10.1073/pnas.0408192102 PMC556237

[18] Bhuvanakantham R, Chong MK, Ng ML (2009) Specific interaction of capsid protein and importin-alpha/beta influences West Nile virus production. Biochem Biophys Res Commun 389: 63–69. 19712667 10.1016/j.bbrc.2009.08.108

[19] Zhang Y, Zhang X, Niu S, Han C, Yu J, et al (2011) Nuclear localization of Beet black scorch virus capsid protein and its interaction with importin alpha. Virus Res 155: 307–315. 21056066 10.1016/j.virusres.2010.10.029

[20] Allen E, Xie Z, Gustafson AM, Sung GH, Spatafora JW, et al (2004) Evolution of microRNA genes by inverted duplication of target gene sequences in Arabidopsis thaliana. Nat Genet 36: 1282–1290. 15565108 10.1038/ng1478

[21] Cai X, Hagedorn CH, Cullen BR (2004) Human microRNAs are processed from capped, polyadenylated transcripts that can also function as mRNAs. RNA 10: 1957–1966. 15525708 10.1261/rna.7135204 PMC1370684

[22] Landgraf P, Rusu M, Sheridan R, Sewer A, Iovino N, et al (2007) A mammalian microRNA expression atlas based on small RNA library sequencing. Cell 129: 1401–1414. 17604727 10.1016/j.cell.2007.04.040 PMC2681231

[23] Cullen BR (2009) Viral and cellular messenger RNA targets of viral microRNAs. Nature 457: 421–425. 19158788 10.1038/nature07757 PMC3074184

[24] Varble A, Chua MA, Perez JT, Manicassamy B, Garcia-Sastre A, et al (2010) Engineered RNA viral synthesis of microRNAs. Proc Natl Acad Sci U S A 10.1073/pnas.1003115107 PMC2895125 20534531

[25] Bartel DP (2004) MicroRNAs: genomics, biogenesis, mechanism, and function. Cell 116: 281–297. 14744438 10.1016/s0092-8674(04)00045-5

[26] Umbach JL, Kramer MF, Jurak I, Karnowski HW, Coen DM, et al (2008) MicroRNAs expressed by herpes simplex virus 1 during latent infection regulate viral mRNAs. Nature 454: 780–783. 18596690 10.1038/nature07103 PMC2666538

[27] Tang S, Bertke AS, Patel A, Wang K, Cohen JI, et al (2008) An acutely and latently expressed herpes simplex virus 2 viral microRNA inhibits expression of ICP34.5, a viral neurovirulence factor. Proc Natl Acad Sci U S A 105: 10931–10936. 18678906 10.1073/pnas.0801845105 PMC2504787

[28] Lo AK, To KF, Lo KW, Lung RW, Hui JW, et al (2007) Modulation of LMP1 protein expression by EBV-encoded microRNAs. Proc Natl Acad Sci U S A 104: 16164–16169. 17911266 10.1073/pnas.0702896104 PMC2042179

[29] Samols MA, Skalsky RL, Maldonado AM, Riva A, Lopez MC, et al (2007) Identification of cellular genes targeted by KSHV-encoded microRNAs. PLoS Pathog 3: e65. 17500590 10.1371/journal.ppat.0030065 PMC1876501

[30] Kalderon D, Richardson WD, Markham AF, Smith AE (1984) Sequence requirements for nuclear location of simian virus 40 large-T antigen. Nature 311: 33–38. 6088992 10.1038/311033a0

[31] Kalderon D, Roberts BL, Richardson WD, Smith AE (1984) A short amino acid sequence able to specify nuclear location. Cell 39: 499–509. 6096007 10.1016/0092-8674(84)90457-4

[32] Goldfarb DS, Gariepy J, Schoolnik G, Kornberg RD (1986) Synthetic Peptides as Nuclear-Localization Signals. Nature 322: 641–644. 3638500 10.1038/322641a0

[33] Lanford RE, Butel JS (1984) Construction and Characterization of an Sv40 Mutant Defective in Nuclear Transport of T-Antigen. Cell 37: 801–813. 6086146 10.1016/0092-8674(84)90415-x

[34] Robbins J, Dilworth SM, Laskey RA, Dingwall C (1991) Two interdependent basic domains in nucleoplasmin nuclear targeting sequence: identification of a class of bipartite nuclear targeting sequence. Cell 64: 615–623. 1991323 10.1016/0092-8674(91)90245-t

[35] Hsieh JC, Shimizu Y, Minoshima S, Shimizu N, Haussler CA, et al (1998) Novel nuclear localization signal between the two DNA-binding zinc fingers in the human vitamin D receptor. Journal of Cellular Biochemistry 70: 94–109. 9632111

[36] Truant R, Cullen BR (1999) The arginine-rich domains present in human immunodeficiency virus type 1 Tat and Rev function as direct importin beta-dependent nuclear localization signals. Mol Cell Biol 19: 1210–1217. 9891055 10.1128/mcb.19.2.1210 PMC116050

[37] Irie Y, Yamagata K, Gan Y, Miyamoto K, Do E, et al (2000) Molecular cloning and characterization of Amida, a novel protein which interacts with a neuron-specific immediate early gene product arc, contains novel nuclear localization signals, and causes cell death in cultured cells. J Biol Chem 275: 2647–2653. 10644725 10.1074/jbc.275.4.2647

[38] Ma H, Zhu J, Maronski M, Kotzbauer PT, Lee VM, et al (2002) Non-classical nuclear localization signal peptides for high efficiency lipofection of primary neurons and neuronal cell lines. Neuroscience 112: 1–5. 10.1016/s0306-4522(02)00044-1 12044466

[39] Cingolani G, Bednenko J, Gillespie MT, Gerace L (2002) Molecular basis for the recognition of a nonclassical nuclear localization signal by importin beta. Mol Cell 10: 1345–1353. 12504010 10.1016/s1097-2765(02)00727-x

[40] Cherezova L, Burnside KL, Rose TM (2011) Conservation of complex nuclear localization signals utilizing classical and non-classical nuclear import pathways in LANA homologs of KSHV and RFHV. PLoS One 6: e18920. 21559489 10.1371/journal.pone.0018920 PMC3084728

[41] Li Y, Zhao L, Wang S, Xing J, Zheng C (2012) Identification of a novel NLS of Herpes Simplex Virus Type 1 (HSV-1) VP19C and Its Nuclear Localization Is Required for Efficient Production of HSV-1. J Gen Virol 10.1099/vir.0.042697-0 22622329

[42] Birbach A, Bailey ST, Ghosh S, Schmid JA (2004) Cytosolic, nuclear and nucleolar localization signals determine subcellular distribution and activity of the NF-kappaB inducing kinase NIK. J Cell Sci 117: 3615–3624. 15252129 10.1242/jcs.01224

[43] Conti E, Kuriyan J (2000) Crystallographic analysis of the specific yet versatile recognition of distinct nuclear localization signals by karyopherin alpha. Structure 8: 329–338. 10745017 10.1016/s0969-2126(00)00107-6

[44] Fontes MR, Teh T, Kobe B (2000) Structural basis of recognition of monopartite and bipartite nuclear localization sequences by mammalian importin-alpha. J Mol Biol 297: 1183–1194. 10764582 10.1006/jmbi.2000.3642

[45] Conti E, Uy M, Leighton L, Blobel G, Kuriyan J (1998) Crystallographic analysis of the recognition of a nuclear localization signal by the nuclear import factor karyopherin alpha. Cell 94: 193–204. 9695948 10.1016/s0092-8674(00)81419-1

[46] Fontes MR, Teh T, Toth G, John A, Pavo I, et al (2003) Role of flanking sequences and phosphorylation in the recognition of the simian-virus-40 large T-antigen nuclear localization sequences by importin-alpha. Biochem J 375: 339–349. 12852786 10.1042/BJ20030510 PMC1223685

[47] Tsang CK, Bertram PG, Ai W, Drenan R, Zheng XF (2003) Chromatin-mediated regulation of nucleolar structure and RNA Pol I localization by TOR. EMBO J 22: 6045–6056. 14609951 10.1093/emboj/cdg578 PMC275436

[48] Haas M, Geldreich A, Bureau M, Dupuis L, Leh V, et al (2005) The open reading frame VI product of Cauliflower mosaic virus is a nucleocytoplasmic protein: its N terminus mediates its nuclear export and formation of electron-dense viroplasms. Plant Cell 17: 927–943. 15746075 10.1105/tpc.104.029017 PMC1069709

[49] Gonzalez I, Martinez L, Rakitina DV, Lewsey MG, Atencio FA, et al (2010) Cucumber mosaic virus 2b protein subcellular targets and interactions: their significance to RNA silencing suppressor activity. Mol Plant Microbe Interact 23: 294–303. 20121451 10.1094/MPMI-23-3-0294

[50] Zhang XR, Henriques R, Lin SS, Niu QW, Chua NH (2006) Agrobacterium-mediated transformation of Arabidopsis thaliana using the floral dip method. Nature Protocols 1: 641–646. 17406292 10.1038/nprot.2006.97

[51] Kaufmann K, Muino JM, Osteras M, Farinelli L, Krajewski P, et al (2010) Chromatin immunoprecipitation (ChIP) of plant transcription factors followed by sequencing (ChIP-SEQ) or hybridization to whole genome arrays (ChIP-CHIP). Nature Protocols 5: 457–472. 20203663 10.1038/nprot.2009.244

[52] Liang XZ, Ding SW, Wong SM (2002) Development of a kenaf (Hibiscus cannabinus L.) protoplast system for a replication study of Hibiscus chlorotic ringspot virus. Plant Cell Reports 20: 982–986.

[53] Zenklusen D, Larson DR, Singer RH (2008) Single-RNA counting reveals alternative modes of gene expression in yeast. Nat Struct Mol Biol 15: 1263–1271. 19011635 10.1038/nsmb.1514 PMC3154325

[54] Li SC, Shiau CK, Lin WC (2008) Vir-Mir db: prediction of viral microRNA candidate hairpins. Nucleic Acids Res 36: D184–189. 17702763 10.1093/nar/gkm610 PMC2238904

[55] Li SC, Pan CY, Lin WC (2006) Bioinformatic discovery of microRNA precursors from human ESTs and introns. BMC Genomics 7: 164. 16813663 10.1186/1471-2164-7-164 PMC1526439

[56] Chen C, Ridzon DA, Broomer AJ, Zhou Z, Lee DH, et al (2005) Real-time quantification of microRNAs by stem-loop RT-PCR. Nucle Acid Res 33: e179. 10.1093/nar/gni178 PMC1292995 16314309

[57] Kim SW, Li Z, Moore PS, Monaghan AP, Chang Y, et al (2010) A sensitive non-radioactive northern blot method to detect small RNAs. Nucleic Acids Res 38: e98. 20081203 10.1093/nar/gkp1235 PMC2853138

