# Supplementary material for: Identification of a Plant Viral RNA Genome in the Nucleus
Source: PLoS One. 2012 Nov 14;7(11):e48736. doi: 10.1371/journal.pone.0048736 (PMC3498252; doi:10.1371/journal.pone.0048736)
Supplement: Table S1 — Primers used in this study. (DOC) [file pone.0048736.s005.doc]

**Table S1 Primers used in this study.**

| **Primers** | **Sequences (5’-3’)** |
| --- | --- |
| **P23F** | **CCGGAATTCATGCTTTCTCAATTGCTTTC** |
| **P23R** | **CGCGGATCCCGGGCGAGTACCCCTGAAA** |
| **P23(1-147)F** | **CCGGAATTCATGCTTTCTCAATTGCTTTCG** |
| **P23(1-147)R** | **CGC GGATCCGCTCATCGCGCATACAG** |
| **P23(46-183)F** | **TATGTGTCGCACCTTTCGCC** |
| **P23(46-183)R** | **CTTCAGGTTCCTCATCAGTGGG** |
| **P23(58-123)F** | **CGGAATTCATGCTTTCGCCGCAGCTG** |
| **P23(58-123)R** | **CGGGATCCCAGCGATGCAAGGATCTCG** |
| **P23(148-627)F** | **CCGGAATTCATGTTGAGGTTGCTGATCCCACTG** |
| **P23(148-627)R** | **CGCGGATCCCGGGCGAGTACCCCTGAAAATC** |
| **H-CP-F** | **CTGAATTCCATGCTGCAGAAGAATG ACC** |
| **H-CP-R** | **GCGGATTC CTAGTTCCTACAGGCCCAC** |
| **qHcVCP(3486-3505)F** | **TGGGATGGAGGTGAAGCAGAA** |
| **qHcVCP(3610-3591)R** | **ACCAAGTGAGTGTGCCTGTG** |
| ***Hib.*act603F** | **ACGAGCAGGAACTGGAGACT** |
| ***Hib.*act734R** | **TGAGTGATGGCTGGAAGAGGA** |
